# The Social Organization of Quality of Life of Older People in Long-Term Care Facilities: An Institutional Ethnography Approach

**DOI:** 10.1177/23333936251324267

**Published:** 2025-03-12

**Authors:** Naomi Hlongwane, Lieketseng Ned

**Affiliations:** 1 Division of Disability and Rehabilitation Studies, Department of Global Health, Faculty of Medicine and Health Sciences, Stellenbosch University, Cape Town, Western Cape, South Africa

**Keywords:** quality of life, long-term care facilities, institutional ethnography, autonomy, meaningful engagement, South Africa

## Abstract

With the growing population in South Africa, there is a need for long-term care facilities. Using institutional ethnography, this study investigates the quality of life for older adults in South African long-term care facilities. Twenty key informants and 10 staff members were purposively sampled across 5 long-term care facilities in Gauteng, South Africa, for participation in in-depth interviews and observations. An analysis of institutional texts was conducted, focusing on legislative frameworks and practices. The findings include three analytic threads, namely: (a) Healthcare Access and Physical Well-Being, (b) Institutional Constraints on Meaningful Engagement, and (c) Efficiency Overriding Privacy and Autonomy. A significant gap exists between legislative policies and actual practices, with older adults seeking more autonomy and decision-making involvement. This institutional ethnography, rooted in the perspectives of older residents and care workers, highlights how long-term care facilities are shaped by regulatory frameworks and institutional ideologies. These frameworks often restrict care workers in fully leveraging their intimate knowledge of residents to address individual needs, as their care work interventions are bound to compliance with the textual and accountability demands of the *Older Persons Act 13 of* 2006.

## Introduction

By 2050, the global population of individuals aged 65 and older is projected to reach 1.5 billion (World Health Organization [WHO], 2021), with the older population in sub-Saharan Africa expected to rise from the 42 million in 2010, to 67 million by 2025 and 163 million by 2050 (Robine, 2021). This is an indication that, globally, there is a demographic transition that is characterized by rapid aging, with an increased life expectancy (Robine, 2021). This transition implies the need to plan for long-term care (LTC) for older people, as the demand for LTC is likely to increase as people age (Kim & Kwon, 2021; Lloyd-Sherlock, 2019). In South Africa, where aging is still largely a family matter, modernization and urbanization are eroding traditional support structures (Cousins et al., 2018). This shift has led to older people considering alternatives, such as LTC facilities (Reinhard, 2019). However, regardless of the level of functioning of the older person when entering these facilities, the transition to LTC is evidenced to have increased stress on quality of life (QoL) (Davies et al., 2023; Srivastava et al., 2021). In a study exploring the impact of physical design on QoL of LTC residents in Canada, it was found that factors such as rigid routines, social isolation, and inadequate personalized care were associated with reduced QoL within LTC facilities (Kervin, 2020). The World Health Organization (WHO) defines QoL as a multifaceted concept influenced by autonomy, health, emotional well-being, and social connections (Van Leeuwen et al., 2019).

Similarly, research conducted in South Africa indicates that QoL is influenced by various multidimensional factors that are underpinned by the WHO definition – aspects such as health, autonomy, relationships, and spirituality, which are critical for older people’s QoL (Anjos et al., 2022; Maharaj, 2020; Van Biljon & Roos, 2015). The South African *Older Persons Act 13 of* 2006 (Republic of South Africa, 2006), aligned with the Madrid Plan of Action (a global framework that addresses the challenges of aging populations), seeks to protect older people by promoting dignity, independence, and participation in society through community-based care and regulated residential facilities (Formosa & Shankardass, 2023; Hoffman & Maloba, 2023). These existing LTC frameworks, and the concomitant structural support they provide, are aimed at promoting the QoL of older people, yet persistent barriers, such as a lack of personalized care tailored to individual needs and preferences, staffing shortages, social isolation, loss of autonomy, inadequate health support, and financial constraints hinder the realization of these aims (Chen et al., 2020; Choe et al., 2018).

This investigation is part of a doctoral study which NH completed in partial fulfillment of her doctoral program, with LN serving as a supervisor. The study was inspired by NH’s previous experience of working in LTC facilities as an occupational therapist, and having witnessed a family member living in a LTC facility. The insights gained as to what was actually happening and what was supposed to happen within the LTC facility created a disjuncture. We wondered, “How is the QoL of older people being constructed and socially organized by the ‘work’ carried out within LTC facilities?” This disjuncture became the central problematic for our study. The problematic is a “puzzle” in the social world, within which disjunctures are explored with the aim of discovering how lives of individuals involved are socially organized to occur as they do (Smith, 2005). The multi-layered complex components that influence the QoL of older people in LTC are not apparent. Therefore, we concluded that an investigation was warranted to uncover both the external and the underlying factors that influence QoL. Our point of entry into the study began at the local unit level, specifically in the Tshwane District government-subsidized LTC facilities in Gauteng, a province of South Africa. We focussed and maintained our investigation from the standpoint of older people, as people’s experiences and knowledge offer hints that enable researchers to trace what happens in the regime of ruling (Rankin, 2017). The research question posed was: What are the daily operations and institutional structures of LTC government facilities and how do they construct the QoL of older people? To explore this, we chose Institutional Ethnography (IE), a critical feminist methodology developed by Dorothy Smith, a Canadian feminist sociologist. This qualitative methodology generates knowledge of how things happen as they do and makes apparent the social organization of peoples’ work (Smith, 2005, 2006).

## Methodology

### Research Approach: Institutional Ethnography (IE)

IE is a research method focused on exploring institutions from the perspective of marginalized and disempowered groups (Smith, 1987). This approach aims to map out ruling relations and understand how institutional powers produce, organize, govern, and coordinate people’s local social environments, knowledge, practices, and activities (Campbell & Gregor, 2002). The primary goal of IE is to uncover “how things are actually put together,” and “how things work” within an institutional framework dominated by these ruling relations (Smith, 2006, p. 1; see also Ion, 2020). Smith defines ruling relations as the broad institutional, managerial, and professional systems that govern and organize society and social life, and these are textually mediated (Smith, 1987). IE researchers uncover and explain how ruling relations regulate, organize, and coordinate people’s behaviors, practices often beyond conscious awareness (Rankin, 2014). Ruling relations are not explicit; rather they are subtly produced through texts, especially when texts are linked to the social organization of power (Campbell & Gregor, 2002). Texts can be written, oral, or visual, examples including films, newspapers, policies, reports, computer programs, social media, patient chart forms, and other institutional documents (DeVault & McCoy, 2006). This approach does not regard an institution as a single entity, but rather as a network of intersecting relations organized around specific functions, such as LTC or legislation (MacDonald, 2017).

In an IE study conducted by Caspar et al. (2016) within LTC, it was found that institutional rules negatively impacted frail older patients and staff, affecting care practices and patient well-being. Similarly, this current study viewed the LTC system as an institutional complex responsible for organizing and regulating LTC delivery and the QoL of care recipients within the LTC system. Further use of IE by researchers has examined how institutional discourses influence informal carers in LTC settings, highlighting themes such as moral obligation, shared care, and task specificity in caregiving (Benjamin, 2011; Caspar et al., 2016; Murphy & Turner, 2017; Øydgard, 2017).

According to Smith, the research begins with the exploration of the everyday life experiences, activities, and perspectives of individuals or groups as they interact within an institutional complex, and then connects these experiences back to the social relations that shape their activities within the complex institution (Grace, 2019).

The focus of this study was on understanding how older people’s QoL is constructed and influenced by the social and ruling relations in LTC facilities.

### Study Setting

Eight government-subsidized LTC facilities for older people, registered with the Department of Social Development (DSD), and situated in a peri-urban community in Tshwane District of Gauteng Province, South Africa were purposively chosen. These facilities were selected due to their reliance on government subsidies, thus reflecting the issue of lack of affordability of LTC for many older persons. These facilities were similar in terms of care provision as outlined by the Regulations regarding older persons that group residents into three categories based on their care needs. Category 1 residents are fully independent and do not require support with daily activities, such as eating, bathing, personal hygiene, using the toilet, and moving around. Category 2 residents need help with these daily tasks, indicating they require assisted living. Category 3 residents are classified as frail and entirely dependent, necessitating 24-hr care due to severe physical or mental conditions that prevent self-care (Republic of South Africa, 2010). These facilities included a combination of these three categories of residents and the accommodation was also arranged based on the care needs. These facilities housed between 50 and 100 residents, staffing included a facility manager, an average of two to three nurses and an average of 10 care workers (care workers are defined in line with the Older Persons Act’s definition of a caregiver: an individual who provides care and support services in settings such as community-based care facilities, residential facilities, or similar establishments, and who has received accreditation through a National Qualifications Framework training qualification suitable for the care of older adults; Republic of South Africa, 2006) per LTC facility, with the majority receiving in-service training. As registered entities with guidelines on care practices and structural requirements, these LTC facilities provide insights into the “work” and ruling relations socially organizing the QoL of older people (Kalideen et al., 2022; Moore & Kelly, 2023). Accessing these closed institutions took 6 months of negotiations with the DSD and facility managers, a process complicated by the sensitivity surrounding research in care facilities following the Life Esidimeni tragedy. This tragedy refers to the deaths of at least 144 psychiatric patients in South Africa between 2015 and 2016, following their transfer from a licensed healthcare facility to unlicensed and under-resourced non-governmental organizations (NGOs) as part of a cost-cutting measure by the Gauteng health department (Durojaye & Agaba, 2018).

### Sampling and Recruitment

The fieldwork, conducted between February 2023 and September 2023, began after receiving ethics approval from the Stellenbosch University Health Research Ethics Committee (reference number: S22/06/105) and institutional permission from the DSD, which registers and governs LTC facilities.

It must be noted that the number of informants in IE research is not specified, the focus instead being on acquiring a sufficient number of participants to represent a varied range of experiences within the institution in order to expose ruling discourses across different times and places (DeVault & McCoy, 2006). However, in order to plan resources, time, and effort effectively, and to ensure that informants were representative of the cultural, social, and institutional context being studied, as well as including diverse perspectives and experiences, NH purposively selected eight LTC facilities to invite their participation in the study, of which five agreed to participate. From these facilities, we recruited 20 older people (standpoint informants). They (standpoint informants) were predominantly older Black individuals, with a majority being female (12 women and 8 men), aged between 62 and 98 years. Many had been residing in the facilities for an extended period, with lengths of stay ranging from 6 months to 7 years. We recruited 10 staff members, including 5 care workers and 5 managers, as secondary informants, who were also predominantly female, ranging in age from 29 to 62 years. Their experience levels varied significantly, from as little as 2 months to over 21 years, while the duration of their employment at the current facilities ranged from 2 months to 8 years.

### Data Collection

All data were collected by NH in 2023, through participant observation, semi-structured interviews with informants, and retrieval and review of forms and relevant documents from the participating LTC facilities. NH identifies as a black woman and an occupational therapist by training. She worked as an occupational therapist for over 7 years in diverse settings, including LTC facilities, where she gained in-depth knowledge of daily operations, health standards, and norms essential to these environments. Her professional experience and understanding of LTC facilities provide her with the expertise needed to conduct research within this setting. Participant observation involved observing each standpoint informant (older person) for a minimum of 60 min as they engaged in the day-to-day activities at the LTC facilities, and shadowing care workers as they carried out their “work,” totaling 30 observational hours. The observations enabled NH to witness the realities of the daily operations within the LTC facilities. The observations were conducted on weekdays over a period of 7 months, depending on the availability of the informants and NH. The observations were recorded using a field note template, guided by Spradley’s (1980) six critical areas:

1. *Space*: What physical space or places are utilized?
2. *Actors*: Who are the people involved in the interaction?
3. *Event/Activities*: What activity is taking place? Describe the environmental context. How is the older person or care worker involved in this activity?
4. *Objects*: Are there any physical objects present during the activity?
5. *Acts*: What actions are being performed and by who? What are people saying (include direct quotes if relevant)?
6. *Feelings*: What emotions are felt/expressed by the individual? What emotions are felt by the researcher in relationship to the interaction?

No personal information was recorded in the field notes. NH read and re-read these after each observation for completeness, and added further details when necessary to ensure nothing was missed. In order to further reveal the problematic,^1^ informant interviews were scheduled and took place in-person in a private room within the LTC facilities. Interviews ranged from 60 to 90 min in length, and were audiotaped, later being transcribed and translated into English, and deidentified to maintain anonymity and protect confidentiality. Interviews were conducted by NH using a semi-structured guide. Interview questions were asked to understand the particular standpoint (i.e., views) of older people’s QoL. Questions were formulated for the purpose of understanding how informants’ QoL was constructed, what care workers considered when making decisions related to their care work and the QoL of older people, and what documents informed their care work and the daily operations of the LTC facilities. The questions sought to comprehensively elucidate the meaning of QoL, focussing on physical well-being, social relationships and interactions, emotional well-being, autonomy and decision-making, safety and security, and recommendations. The interviews started with questions such as: “Could you please take me through your typical day?” Some questions were a follow-up on what was observed, or sought clarity regarding documents and forms used during observation, such as, “I saw you doing this during leisure time; can you tell me what was going on?” Probes were used to encourage the informants to further share their stories and provide descriptions of their everyday experiences. To identify the ruling that organized the QoL of older people, NH interviewed five managers. Smith (1987) recognizes how society is text-based, and the activation of certain, overarching institutional texts, or “boss texts,” mediates people’s work and influences people’s understanding of their experiences. NH paid attention to all texts used during the observation, usually clinical or health authority documents, or documents mentioned during interviews, or regulatory administrative documents. Eventually, a “chain” of text and process was constructed by attending to the connections that both the standpoint informants (older people) and secondary informants (care workers and managers) revealed during their interviews.

### Analytic Process and Mapping

Data collection and data analysis occur simultaneously during an IE study (Campbell & Gregor, 2002). Data analysis entails iterative reading and re-reading of data collected from field notes, interviews, and key documents. The focus of data analysis was on explicating how the daily operations and institutional structures socially organized the QoL of older people. Rankin (2017) cautions institutional ethnographers to resist looking for categories, patterns, or themes in their data. Rather, the process of analysis is “rather like grabbing a ball of string, finding a thread, and then pulling it out” (DeVault & McCoy, 2002, p. 755). NH immersed herself in the data, beginning with field notes obtained from participant observations and reading and re-reading interview transcripts. She then imported the interview transcripts into Dedoose Software, using the memo function to highlight terms or concepts imported from other texts and discourses, and adding comments.

**Figure 1.**
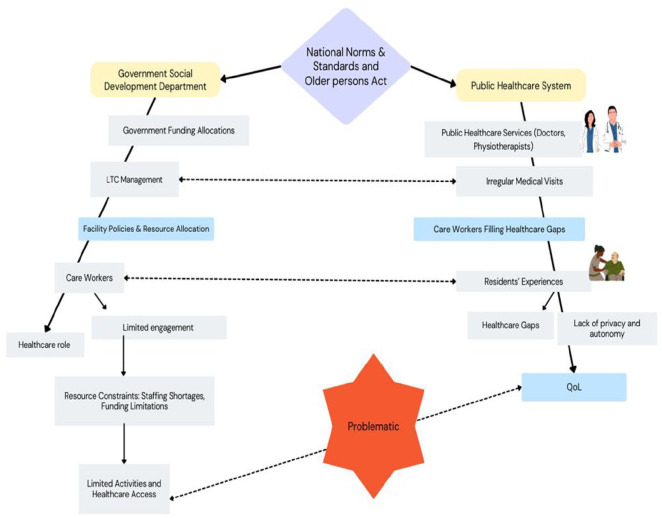
Mapping quality of life.

Once connections began to be constructed in the analysis, we (authors) started to link pieces of the data together by using analysis techniques unique to IE, including indexing (Table 1) and mapping (Figure 1; Rankin, 2017). We used indexing to create a cross-reference for linked process and texts. Development of the links was based on questions such as: What aspect of QoL is the informant describing? How is this standpoint informant’s QoL connected to the work carried out by care workers? What aspects of the institutional dimension is this informant describing? How is QoL related to these aspects? (McCoy, 2006). NH created folders for terms that were frequently used by informants, and indexed all data (written descriptions of process and texts) related to these terms in the folders. For example, when both the standpoint informants and secondary informants talked about “privacy” and “shared spaces,” they frequently talked about “autonomy and privacy” (a component associated with QoL as per the WHO definition of QoL), so NH created an “autonomy and privacy” folder. Next, she put quotes from interviews and documents related to autonomy and privacy in that folder. The contents of the folders then became the ends of the analytical threads NH followed to untangle how QoL was organized. NH then used mapping to display what was happening and track ruling relations (Rankin, 2017). Specifically, the mapping work included a diagram that plotted the various texts and processes that organized QoL in order to show and describe “how things work.

**Table 1.** Example of indexing.

Observation	Interview data	Institutional trace	Indexing
Care workers attending to older people’s needs such as giving of medication, wound care, and taking vitals. Furthermore there are more care workers than nursing staff	Well, since I’ve been here, I haven’t seen the doctor, even the social worker or physiotherapist. (Care worker, six months’ employment duration.)	The Older Persons Act and National Norms and Standards mandate 24-hr care for frail older persons, including those with dementia and related conditions, as well as rehabilitation services. Regulations from the Department of Social Development require staffing in care facilities to consist of at least 33% registered nurses (RNs), with up to 50% replaceable by enrolled nurses (ENs), resulting in 16.5% RNs and 16.5% ENs. The remaining 66% may be enrolled nursing assistants (ENAs), with up to 50% replaceable by caregivers, resulting in 33% ENAs and 33% caregivers	What is being prescribed by the Older Persons Act, the National Norms and Standards and the regulations for older persons is not taking place in reality as there is reduced access to healthcare and care workers are taking on the responsibility of nurses, with care workers exceeding the prescribed ratio and skill mix

### Trustworthiness

To ensure the trustworthiness of this study, NH maintained prolonged engagement with the informants to ensure a comprehensive understanding of the context. In addition, through persistent observation, and the use of multiple data sources and methods of data collection, we were able to ensure triangulation of data. We extracted quotes from the informants’ transcripts to support the claims that are being made, in keeping with the IE tenet of supporting conformability. We focused on the empirical excerpts – those quotes that revealed traces of the informants’ material world, those aspects of things happening. We also maintained a detailed audit trail that included the storage of all data in a qualitative software program to ensure dependability. Although institutional ethnographers are not interested in establishing the transferability and generalizability of their data, they are interested in describing and generalizing practices that cut across several settings (DeVault & McCoy, 2002). The enlistment of the five sites allowed us to discover the ruling relations that cut across sites, including government standards and accountability practices that governed not only the sites where data were gathered, but also other LTC sites where workplace practices are socially organized in similar ways.

## Findings

The findings presented here detail the “disjuncture” between the QoL of older people within LTC facilities and ruling representations of how LTC facilities were organized that became obvious during the observations. What became apparent was the disconnection between the goals of how LTC facilities are supposed to enhance the QoL of older people and the actual experiences of QoL of older people, and how the care workers’ work organized the QoL. This disjuncture became the problematic of this study, which we further explored through analytical threads and mapping.

In this section of the paper, we begin with a critical analysis of the social organization of QoL under three analytic threads, which include: (a) Healthcare Access and Physical Well-being, (b) Institutional Constraints on Meaningful Engagement, and (c) Efficiency Overriding Privacy and Autonomy. Each of these linked analytical threads provide the basis upon which we assert that LTC facilities’ daily activities are organized, and our findings show that QoL is shaped by complex institutional guidelines and work processes. In the following presentation of findings, older people are referred to as residents.

In an IE, the findings and discussion are methodologically and theoretically linked (Benjamin et al., 2016; Caspar et al., 2020). Therefore, the findings section of this article includes both the analytical threads, as well as related discussion regarding the ways the QoL of older people might be considered in light of the goal of IE. Importantly, although relevant literature is incorporated, the intent is to demonstrate the social organization of QoL of older people, rather than comparing it to the findings of other researchers (Benjamin et al., 2016; Campbell & Gregor, 2002; Caspar et al., 2020; Smith, 1987).

### Analytical Thread 1: Healthcare Access and Physical Well-Being

Observations of residents revealed that, in some instances where healthcare professionals were not available, they relied on the care workers to attend to their medical needs, and this included offering physical exercise. The accounts from residents’ interviews clarified these observations as residents indicated that they are receiving inadequate access to healthcare professionals, such as doctors, physiotherapists, and social workers within the facilities. For example, one resident shared how healthcare services had diminished over time:

Before, the students from Medunsa^2^ used to come here. They would help us with exercising every morning. We started with this sister who was studying at Medunsa but then she got a job at Garankuwa, at George Mukhari. Then these ones came and trained us. And they also left. Now there is this girl, now she is on leave, she is a nurse. She is the one who comes every morning. When we get there, we will stretch and jump up and down. Now that she is not here, you only stand up when you go to the toilet. (85-year-old male, resident for 3 years)

This statement illustrates the discourses in the provision of physical training and healthcare services, which are recognized as core components of LTC under the – The Older Persons Act and the National Norms and Standards mandate the provision of “24-hour care and support services for frail older persons requiring special attention, care, and supervision, including services for those suffering from dementia and related conditions, as well as rehabilitation services.” Additionally, older persons must have “access to opportunities that promote their optimal level of physical well-being.” A review of the shift scheduling roster – which was implemented to provide 24-hr care – at one facility revealed a block shift approach with fixed routines spanning 2 weeks. However, there was an imbalance in the staff skill mix, which may explain instances of care workers performing tasks beyond their designated scope. The lack of continuous support, such as regular visits from healthcare professionals, creates a significant gap in the care received by residents and contradicts what is expected as indicated by the institutional text. Another resident voiced frustration with the inadequacy of physical exercise programs, stating:

Two minutes and we are done. They don’t do serious exercises. Because they should check who can do what. And then we can compete with each other and say who can do better than who. And you become active. So here it is sleep, wake up, bath and eat. That is the routine. (75-year-old female, resident for 1 year)

These observations emphasize how institutional arrangements fail to fulfill the expectations outlined in policies, leading to a diminished QoL for older people.

Observations of care workers’ “work” revealed that some care workers were responsible for giving medication to residents after breakfast. Care workers were observed having a list of the names of those requiring medication, the type of medication, and number of pills. Care workers also acknowledged the limited access to healthcare services. One care worker reflected on the gap in medical support, stating:

Well, since I’ve been here, I haven’t seen the doctor, even the social worker or physiotherapist. Well, they sometimes ask me, “My child, help me here, massage me there.” That is what I do. (Care worker, six months’ employment duration)

This care worker’s experience reflects the institutional reality for care workers who are relied on to fill the gaps in medical care, despite their limited training. The reliance on care workers for healthcare tasks, due to the absence of medical professionals, further highlights the institutional challenges that compromise the physical well-being of older people in LTC facilities. A manager in an interview, when asked about this practice, revealed that this was beyond the training scope of care workers, however, due to staff issues and lack of support from the Department of Health, the more experienced care workers assist in this task.

In mapping this analytical thread (see Table 1 below), the findings evidence that the poor staffing and resource allocation of LTC facilities led to work demands on care workers that was beyond their training and not part of their daily work organization. This meant poor access to adequate healthcare and physical activity for older people, and decreased QoL. The discrepancies between legislative text – The Older Persons Act and National Norms and Standards mandate 24-hr care for frail older persons, including those with dementia and related conditions, as well as rehabilitation services. Regulations from the Department of Social Development require staffing in care facilities to consist of at least 33% registered nurses (RNs), with up to 50% replaceable by enrolled nurses (ENs), resulting in 16.5% RNs and 16.5% ENs. The remaining 66% may be enrolled nursing assistants (ENAs), with up to 50% replaceable by caregivers, resulting in 33% ENAs and 33% caregivers – which advocate for continuous care and actual practices within LTC facilities compromises the physical well-being of residents, for whom regular, comprehensive medical attention is neglected.

### Analytical Thread 2: Institutional Constraints on Meaningful Engagement

Observations and interviews with informants (older people and care workers) consistently revealed that engaging in a variety of social and leisure activities was not considered to be integral to the routine and wellness-enhancing daily care of residence. As one resident described, “There’s nothing that we do. We just sit like this. We eat, we just sit by the sun until we go to the shade” (82-year-old male, 6 years in the facility).

This description captures the sense of passivity and isolation that residents feel due to the limited availability of recreational or social activities. Institutional planning evidently prioritized operational efficiency over individual needs, with care worker observations revealing a focus on a limited range of core resident care needs (e.g., bathing, dressing, and eating). The review of the duty rosters highlighted this issue, as the rosters listed staff members by name and allocated duties based on their designation, including care provision, meal preparation, and bathing. However staff were not assigned to facilitate dedicated social or recreational activities. However, religious activities were a dominant form of engagement, especially in facilities with church affiliations, which some residents found spiritually fulfilling. For example, a 74-year-old female resident noted, “It frees my spirit.. . . I’m doing quite alright here and my family is doing alright at home too,” reflecting the importance of facilitating residents’ engagement in religious activities for their well-being.

However, beyond religious services, there was a marked absence of other forms of meaningful activities. A common desire among the residents was for opportunities to engage in games and interactive activities. An 85-year-old woman, who had been in the facility for 7 years, reminisced, “I remember some time back there were balls that we would pass around.. . . But now, ours is sit, eat and sleep.”

This lack of active programming underscores the institutional limitations that prioritize care routines over holistic engagement where social and recreational participation is considered external to the core operations of the LTC facilities, resulting in residents feeling mentally and physically unstimulated. Care workers, too, acknowledged the lack of structured activities, with one worker stating, “I wish there was more activities . . . not doing anything can make them sick.” This statement reflects both an awareness of the impact of inactivity on residents’ health, and frustration with the lack of resources and funding to implement more robust activity programs. Certainly, the social organization of participating in social and leisure activities was confirmed in an interview with a manager who described her awareness that social and leisure activities are a requirement of LTC services, and that an agreement with a University to have their occupational therapy students do their in-service training at the facility had failed after a few months. Another manager described how, “My budget does not allow for me to buy balls and board games. I rely on donations and volunteers to keep the older people busy.”

In mapping this analytical thread (see Figure 1 below), the findings evidence that the support required for participation in social and leisure activities was not built into the standard care for LTC residents, despite legislation such as the Older Persons Act, and national norms and standards mandating that residents should participate in organized activities, including but not limited to reading, radio and TV, and religious and cultural activities, thus promoting well-being. We argue that when meaningful activity is organized as an add-on program, separately funded, provided by experts and governed and monitored by people external to the daily routine in LTC, it becomes a ruling relation that disrupts everyday knowledge about what is needed in each LTC facility and for each LTC resident. The various LTC standards (stipulated within the National Norms and Standards and Older Persons Act), along with monitoring and accountability practices that are to be conducted by the Department of Social development as routine audit of LTC facilities and the quarterly reports that need to be submitted by the facility to the Department of Social Development, serve as powerful coordinators that generate what Smith (2005) describes as an “institutional discourse” (p. 225) about what occurs within LTC facilities. This discourse prioritizes certain aspects of people’s actions that can be held accountable within its framework, while overlooking the realities of the actual lived experience and needs of residents in LTC (Smith, 2005, p. 225). This gap between policy ideals and actual practice results in a diminished QoL for older residents, who experience social isolation and a lack of physical and mental stimulation.

### Analytical Thread 3: Efficiency Overriding Privacy and Autonomy

Observations of older people as they prepared to take afternoon naps as part of their daily activities revealed that older people shared sleeping spaces and lacked privacy and autonomy. This impactful revelation was further explored in an interview with a resident who expressed frustration with the lack of privacy, particularly in relation to romantic relationships, stating, “As you can see, there is no privacy here.. . . It’s not possible.” This exemplifies how institutional organization shapes residents’ daily lives, with their personal preferences and desires being subordinated to the logistical needs of the facility. This insight was then probed with a care worker from the same facility, who further added that residents feel self-conscious when bathing in the presence of others: “There is a granny, when she takes a bath she has no self-confidence.. . . She needs privacy.” Despite recognizing the residents’ need for privacy, care workers are constrained by the institutional structure, which prioritizes cost-effectiveness over individualized care. This was starkly exemplified by the revelation that residents in one facility not only shared physical spaces, but even personal items, due to lack of funds, as described by a care worker: “It’s so painful when we bathe them. They have to share a facecloth. Even when I talk about it, I become emotional.”

It would later be clarified by a manager through interview that institutional practices, such as shared rooms and the segregation of residents based on frailty and care needs, are framed as necessary for operational efficiency, as she referred me to the national norms and standards that stipulate that those residents under frail care can be assigned to a room with a maximum of four residents per room. Despite these stipulations, however, one room had over 20 older people sleeping in it, exemplifying gross disparities between norms and standards espoused by the manager interviewed, and what was witnessed as the lived reality of the residents of that LTC facility.

This violation of privacy, driven by resource constraints and the prioritization of efficiency, reflects how the implementation of policies is often undermined by the realities of institutional management. Residents are also largely excluded from decision-making processes, which further erodes their sense of autonomy. This was demonstrated during my observations of a lunch time during which a resident requested a cup of tea instead of the soup and bread brought to her by a care worker. The care worker responded, “You can’t choose what you want to eat you know. We have a standard meal plan here.” This lack of autonomy was probed in the interview with the resident who explained: “I would like to be involved so I can speak up on what they can improve.. . . They don’t involve us at all.” This focus on efficiency also affects the emotional well-being of residents, as noted by a care worker who expressed a desire for more resources to support resident autonomy: “They must live a homey life. The challenge is, we can’t give them that experience in full because of limited funds.” This lack of autonomy illustrates how ruling relations, shaped by institutional structures, limit residents’ ability to influence the conditions of their own care, which is contrary to the rights enshrined in institutional texts; The Older Persons Act emphasizes the importance of respecting the rights of older persons in residential facilities. In addition to the rights outlined in the Bill of Rights, older persons have the right to participate in social, religious, and community activities of their choice, as well as the right to privacy and to keep and use personal possessions. Violations of these rights, such as repeated breaches of privacy, may constitute psychological abuse and a violation of the older person’s right to privacy under section 16 of the act (Republic of South Africa, 2006).

In mapping this analytical thread (see Figure 1 below), findings evidence that these institutional decisions are not isolated, but are part of broader ruling relations – the systems of governance that structure life in institutional settings. The institutional constraints on funding and resources directly shape the day-to-day realities of both care workers and residents, limiting the facility’s ability to create an environment that respects the personal dignity and autonomy of its residents. These ruling relations are mediated through policies that focus on managing resources, staff allocation, and the physical layout of the facility to ensure smooth operations, often without regard for the personal experiences of the individuals residing there. Although institutional texts, such as the Older Persons Act and the Bill of Rights, affirm the right to privacy and personal autonomy for older people, the lived experiences of residents suggest a significant disjuncture between policy and practice. Through the lens of IE, the focus on efficiency within LTC facilities is revealed to be a ruling relation that systematically organizes care in ways that conflict with residents’ rights to privacy and autonomy, negatively impacting QoL.

## Discussion

The empirical links between the daily operations, LTC standards, and the QoL of older people provide a nuanced understanding of what has been documented regarding the QoL of older people following an older person’s admission to government-subsidized LTC facilities.

The findings from this study describe the daily operations of LTC facilities, and how “work” is organized and shapes QoL for older people. The findings show how inactivity is socially organized, and the promotion of meaningful activity is not considered to be integral or necessary to daily routines. Meaningful activity is regarded as an add-on, a program that is parcelled out and designated as specialized work in the purview of occupational therapists. The new finding this research contributes is an analytical understanding of what has previously been discussed in the literature as barriers to meaningful engagement and QoL in LTC settings. A scoping review of patterns of behavior found older people living in care homes spend between 71% and 98% of their waking hours engaged in sedentary activities, such as watching TV, using a computer, and talking to others (Leung et al., 2021). However, this limited range of activities does not reflect older people’s interest patterns, as evidenced in a study conducted in South African LTC facilities that found that older people were most interested in musical, social, free time, and religious or cultural activities (Plastow et al., 2024). The current study therefore suggests that the inclusion of key identified activities of interest within the LTC program embedded within the “work” conducted by care workers, and included in the funding model for LTC, could translate into the meaningful engagement of activities by older people, and thus improve their QoL. Furthermore, supporting the findings of this study, recent studies evidence that engagement in physical activity has a positive impact on health-related QoL, particularly during significant life events, and that participation in organized social activities in LTC can effectively slow down the decline in QoL over time for older people (Boamah et al., 2021; Conboy Russo, 2022). Overall, promoting meaningful activities and engagement is essential for enhancing the well-being and QoL of older people in LTC settings (Magistro et al., 2021).

Suboptimal opportunities for residents’ physical activity and access to adequate healthcare services are systematically organized within a broader set of interconnected practices, as revealed in our findings. Our observations and interview data establish key connections between healthcare standards, procedures related to providing basic health services, participation in physical activity, and the institutional use of occupational therapy services that are external to the “work” of the LTC, showing how these factors contribute to the QoL of older people. These findings reveal how features of daily operations in LTC are discursively cemented by narrow ideological constructions of person-centered approaches (Schweighart et al., 2022). These care approaches shape the perceptions of what residents need, and what care workers and managers believe. These ideas are further solidified through checklists, guidelines and standards representing a textual version of what is perceived to be achieved in LTC facilities.

Our research suggests that these established features of life in LTC combine to create a socially organized environment of inactivity and lack of autonomy. Privacy and autonomy emerged as critical issues within the LTC facilities. Older people share rooms, which leads to discomfort and a lack of personal space, particularly for those who require privacy during personal care activities. The sharing of basic items, such as facecloths, further infringes on personal dignity and autonomy. These conditions contrast starkly with the rights outlined in the Older Persons Act, which stipulate the right to privacy and the maintenance of personal possessions. The insights shared by older people reveal a sense of disempowerment and a desire for greater involvement in decisions affecting their living conditions. Residents are daily confronted by barriers to the benefit they are entitled to derive from the documented advantages of being more active and having a sense of identity and autonomy (Hillcoat-Nallétamby, 2014).

Research consistently suggests that perceived autonomy in LTC environments positively influences overall QoL, with key factors such as independence, physical and mental competence, and family support playing crucial roles (Moilanen et al., 2021). A study conducted in Uganda and South Africa similarly found that difficulties in performing activities of daily life have been significantly associated with poorer health outcomes and QoL among older people (Yaya et al., 2020). In Taiwan, Li-Hsing and Chia-Chan (2020) examined the relationship between functional capacity and life satisfaction in older peoples living in LTC facilities. Their findings indicate that autonomy mediates this relationship and impacts life satisfaction. They suggest that facility managers and staff develop self-support programs that encourage older people to participate in physical activities and maintain autonomy (Li-Hsing & Chia-Chan, 2020). Therefore, fostering autonomy and enabling older people to make choices regarding their care and daily activities can contribute to enhanced health outcomes and improved QoL in residential care settings.

This study highlights critical challenges faced by a resource limited context such as South Africa’s LTC sector, including poor staffing, inadequate training, and financial constraints. Evidence shows that the LTC workforce is insufficiently trained to meet the complex needs of the aging population (Kelly et al., 2019; Mapira et al., 2019; Naidoo & Van Wyk, 2019). Further insights from this study show that staff shortages often compel care workers to perform tasks beyond their formal roles, adversely affecting the quality of care and underscoring the urgent need for workforce development. A 2010 national survey of 405 LTC facilities revealed that only 25% of staff were aware of official care standards. This gap in knowledge was reflected in the Life Esidimeni tragedy, where systemic failures led to the unlawful deaths and forced transfer of residents (Makgoba, 2017). Furthermore, the Department of Social Development (DSD) found that care workers’ qualifications are not recognized for career advancement, leading to job dissatisfaction and the perception of care work as lacking career progression. Despite advocacy by the African Union for improved workforce policies, regulatory frameworks such as the Older Persons Act and the Policy on Social Service Practitioners remain inadequately implemented. This study underscores the urgent need to prioritize the well-being of older people by strengthening the workforce and exploring alternative funding models for LTC.

There are no simple solutions. Calls for an amendment to the Older Persons Act and the National Norms And Standards are unlikely to disrupt the way that guidelines and Acts enter and organize a local setting. The major recommendation arising from this IE is directed to people at the level of decision-making to find informal, locally driven ways to use the knowledge that care workers have about residents, and the work practices and direct inputs from residents on the factors that shape their QoL, to create a LTC policy that is co-created and addresses the needs of older people (Hlongwane, 2024). The creation and implementation of policies and systems for LTC of older adults have been largely overlooked in the Southern African region due to competing priorities and a strong preference for family- and community-based care. This has led to governments taking minimal responsibility for supporting formal care settings, while also failing to sufficiently strengthen families’ ability to provide adequate care (WHO, 2017).

## Conclusion

In conclusion, this IE, rooted in the perspectives of older residents and care workers, highlights how LTC facilities are shaped by regulatory frameworks and institutional ideologies. These frameworks often restrict care workers from fully leveraging their intimate knowledge of residents to address individual needs, as their work must adhere to the textual and accountability demands of the Older Persons Act. Our findings suggest that to promote genuine QoL improvements, LTC facilities would benefit from forming internal coalitions comprizing residents, care workers, nurses, and managers. These coalitions could examine operational practices without being constrained by the current bureaucratic standards, fostering a more flexible approach to QoL. This study, through first-hand narratives, exposes how LTC work practices are deeply intertwined with accountability requirements that often hinder personalized care. A coalition approach, informed by this research, could support LTC stakeholders in reinterpreting care standards, fostering meaningful activities, and reducing excessive standardization. This study highlights the need for an expanded evidence base to help researchers and policymakers challenge generalized QoL metrics and advocate for targeted, evidence-based interventions. Achieving this goal requires cross-national and cross-disciplinary collaboration, increased research funding, and comprehensive policy frameworks that integrate LTC into national health, social protection, and care systems, while acknowledging the vital role of family caregivers. Sustainable funding models are essential, requiring a shift from sole reliance on government funding to incorporating private contributions and partnerships. Additionally, robust frameworks are needed to guide effective staffing models in LTC facilities. This approach could adequately prepare for an aging population by engaging a wide range of stakeholders and utilizing resources from the private sector. Ultimately, this research lays the groundwork for reimagining QoL in LTC, advocating for practical, resident-focused improvements that enhance daily life within these facilities.

## Notes

1. To return to Smith’s (2005) definition of the “problematic,” provided in the introductory section of this article, the problematic is a “puzzle” in the social world, within which disjunctures are explored with the aim of discovering how lives of individuals involved are socially organized to occur as they do. The central problematic for our study was based on the question, “How is the QoL of older people being constructed and socially organized by the ‘work’ carried out within LTC facilities?”

2. Previously known as Medical University of South Africa and later as MEDUNSA campus of the University of Limpopo, as of 2015, known as Sefako Makgatho Health Sciences University, a medical university in Ga-Rankuwa, Gauteng Province, South Africa.
